# (5-Benzoyl-3,6-dimeth­oxy­naphthalen-2-yl)(phen­yl)methanone

**DOI:** 10.1107/S160053681101508X

**Published:** 2011-04-29

**Authors:** Yuichi Kato, Atsushi Nagasawa, Kosuke Sasagawa, Akiko Okamoto, Noriyuki Yonezawa

**Affiliations:** a Department of Organic and Polymer Materials Chemistry, Tokyo University of Agriculture and Technology, 2-24-16 Naka-machi, Koganei, Tokyo 184-8588, Japan

## Abstract

The asymmetric unit of the title compound, C_26_H_20_O_4_, contains two independent conformers. The aromatic rings of the aroyl groups are twisted with respect to the naphthalene ring systems to form dihedral angles of 66.58 (6) and 66.45 (6)° in one conformer, and 75.00 (7) and 81.17 (6)° in the other conformer. The crystal packing is stabilized by weak inter­molecular C—H⋯O hydrogen bonds and by C—H⋯π inter­actions.

## Related literature

For information on the electrophilic aromatic substitution of naphthalene derivatives, see: Okamoto & Yonezawa (2009 ▶). For the structures of closely related compounds, see: Kataoka *et al.* (2010 ▶); Kato *et al.* (2010 ▶, 2011 ▶); Nakaema *et al.* (2008 ▶); Nishijima *et al.* (2010 ▶); Watanabe *et al.* (2010 ▶).
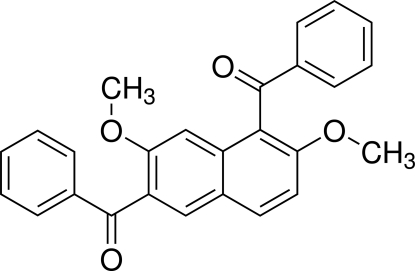


## Experimental

### 

#### Crystal data

C_26_H_20_O_4_
*M* *_r_* = 396.42
Triclinic, 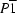

*a* = 8.42828 (15) Å
*b* = 12.5953 (2) Å
*c* = 20.0578 (4) Å
α = 96.222 (1)°
β = 99.688 (1)°
γ = 102.727 (1)°
*V* = 2023.76 (6) Å^3^
*Z* = 4
Cu *K*α radiation
μ = 0.71 mm^−1^
*T* = 193 K
0.60 × 0.40 × 0.10 mm

#### Data collection

Rigaku R-AXIS RAPID diffractometer
Absorption correction: numerical (*NUMABS*; Higashi, 1999 ▶) *T*_min_ = 0.677, *T*_max_ = 0.933
32431 measured reflections
7263 independent reflections
5957 reflections with *I* > 2σ(*I*)
*R*_int_ = 0.031

#### Refinement

*R*[*F* ^2^ > 2σ(*F* ^2^)] = 0.039
*wR*(*F* ^2^) = 0.115
*S* = 1.08
7263 reflections
546 parameters
2 restraints
H-atom parameters constrained
Δρ_max_ = 0.27 e Å^−3^
Δρ_min_ = −0.21 e Å^−3^

### 

Data collection: *PROCESS-AUTO* (Rigaku, 1998 ▶); cell refinement: *PROCESS-AUTO*; data reduction: *CrystalStructure* (Rigaku, 2010 ▶); program(s) used to solve structure: *IL MILIONE* (Burla *et al.*, 2007 ▶); program(s) used to refine structure: *SHELXL97* (Sheldrick, 2008 ▶); molecular graphics: *ORTEPIII* (Burnett & Johnson, 1996 ▶); software used to prepare material for publication: *SHELXL97*.

## Supplementary Material

Crystal structure: contains datablocks I, global. DOI: 10.1107/S160053681101508X/rz2585sup1.cif

Structure factors: contains datablocks I. DOI: 10.1107/S160053681101508X/rz2585Isup2.hkl

Supplementary material file. DOI: 10.1107/S160053681101508X/rz2585Isup3.cml

Additional supplementary materials:  crystallographic information; 3D view; checkCIF report

## Figures and Tables

**Table 1:** Hydrogen-bond geometry (Å, °) *Cg*1 and *Cg*2 are the centroids of the C19–C24 and C4–C9 rings, respectively.

*D*—H⋯*A*	*D*—H	H⋯*A*	*D*⋯*A*	*D*—H⋯*A*
C15—H15⋯O3^i^	0.95	2.57	3.5191 (19)	176
C25—H25*C*⋯O4^ii^	0.98	2.56	3.348 (2)	138
C51—H51*C*⋯O8^iii^	0.98	2.47	3.371 (2)	152
C3—H3⋯*Cg*1^iv^	0.95	2.59	3.416 (11)	145
C14—H14⋯*Cg*2^v^	0.95	2.86	3.578 (9)	133

Symmetry codes: (i) 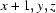; (ii) 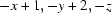; (iii) 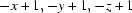; (iv) 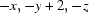; (v) 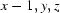.

**Fig. 1.**
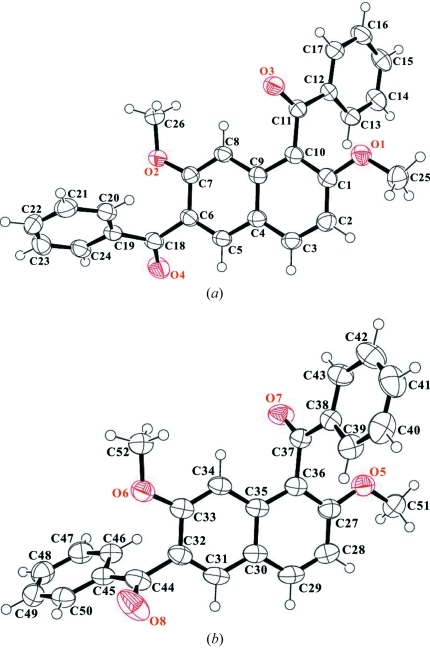
The molecular structure of conformers (I) and (II). Displacement ellipsoids are drawn at the 50% probability level.

**Fig. 2.**
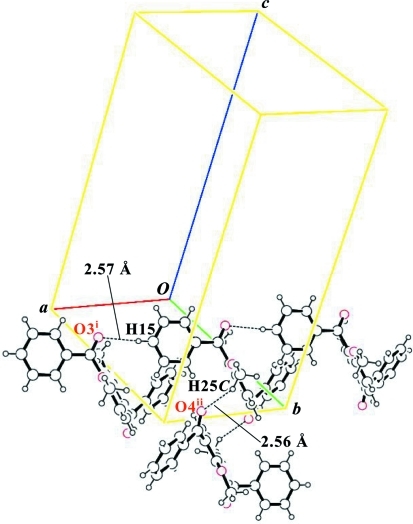
Intermolecular C—H···O interactions in conformer (I) [symmetry code: (i) -x+3/2, y-1/2, z; (ii) -x+1, -y+2, -z].

**Fig. 3.**
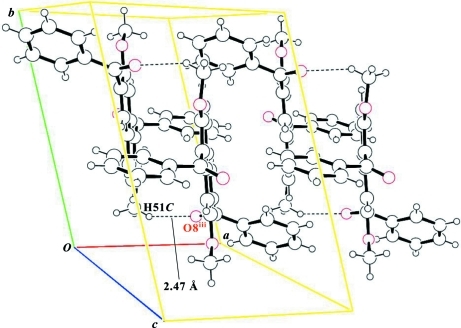
Intermolecular C—H···O interactions in conformer (II) [symmetry code: (iii) -x+1, -y+1, -z+1].

**Fig. 4.**
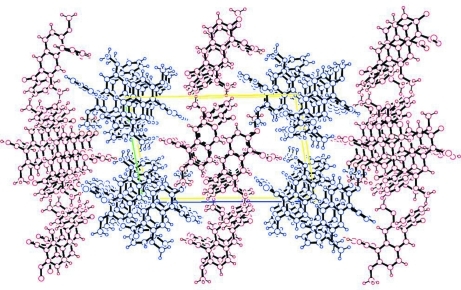
The alignment of the molecules in the crystal structure, viewed along the a axis [conformer (I) is blue, conformer (II) is red].

## supplementary crystallographic information

### Comment

In the course of our study on electrophilic aromatic aroylation of 2,7-dimethoxynaphthalene, *peri*-aroylnaphthalene compounds have proven to be formed regioselectively with the aid of suitable acidic mediators (Okamoto & Yonezawa, 2009). Recently, we have reported the crystal structures of several 1,8-diaroylated naphthalene homologues exemplified by 1,8-bis(4-aminobenzoyl)-2,7-dimethoxynaphthalene (Nishijima *et al.*, 2010) and 1,8-dibenzoyl-2,7-dimethoxynaphthalene (Nakaema *et al.*, 2008). The aroyl groups at the 1,8-positions of the naphthalene rings in these compounds are connected in an almost perpendicular fashion. In this course, the crystal structures of 1-monoaroylated naphthalene compounds and the β-isomers of 3-monoaroylated compounds have been also clarified such as 1-benzoyl-2,7-dimethoxynaphthalene (Kato, *et al.*, 2010), 1-(3-nitrobenzoyl)-2,7-dimethoxynaphthalene (Kataoka *et al.*, 2010), 3-benzoyl-2,7-dimethoxynaphthalene (Kato *et al.*, 2011), and (3,6-dimethoxy-2-naphthyl)(4-fluorophenyl)methanone (Watanabe *et al.*, 2010). 1-Aroylated naphthalene compounds have been revealed to have essentially the same non-coplanar structure as the 1,8-diaroylated naphthalenes. 3-Substituted aroylnaphthalene compounds are generally regarded to be thermodynamically more stable than the corresponding 1-positioned isomeric molecules, with the aroyl groups connected to the naphthalene rings in a moderately twisted fashion. As a part of our continuous study on the molecular structures of this kind of homologous molecules, the crystal structure of title compound, a 1,6-dibenzoylated naphthalene derivative, is discussed in this paper.

There are two independent conformers in the asymmetric unit of the title compound. The conformers, labeled (I) and (II), are shown in Fig. 1. Each conformer has essentially the same non-coplanar structure, the main difference consisting in the dihedral angles formed by the benzene rings with the naphthalene ring systems. Conformer (II) shows a larger dihedral angle for the benzene ring of the aroyl group at 6-position than that of the benzene ring of the aroyl group at 1-potision [81.17 (6) and 75.00 (7)°], whereas very similar dihedral angles are observed for conformer (I) [66.45 (6) and 66.58 (6)°]. These  angles could be compared with those reported for related 1- and 3-monoaroylated naphthalenes, *e. g.* (2,7-dimethoxynaphthalen-1-yl)(phenyl)methanone (75.34 (7), 86.47 (7) and 76.55 (6)°; Kato *et al.*, 2010) and (3,6-dimethoxynaphthalen-2-yl)(phenyl)methanone (68.32 (5)°; Kato *et al.*, 2011). The torsion angles between the carbonyl groups and the naphthalene ring of conformer (I) are 116.90 (14) (C1—C10—C11—O3) and 48.7 (2)° (C5—C6—C18—O4), those of conformer (II) are 106.70 (17) (C27—C36—C37—O7) and 73.7 (2)° (C31—C32—C44—O8). In the crystal structure, the molecular packing is stabilized mainly by weak two intermolecular C—H···O hydrogen bonds in conformer (I) (Table 1, Fig. 2). Moreover, a C—H···O hydrogen bond between the hydrogen atom of a 2-methoxy group and the oxygen atom of a carbonyl group is observed in conformer (II) (Table 1, Fig. 3). The crystal structure is further stabilized by C—H···π interactions (Table 1). In the crystal structure, conformer (I) and (II) are alternately piled up along *a* axis as shown in Fig. 4.

### Experimental

To a 50 ml flask, benzoyl chloride (3.2 mmol, 350 mg), aluminium chloride (3.4 mmo1, 450 mg) and methylene chloride (2.5 ml) were added and stirred at 273 K. To the reaction mixture thus obtained, was then added 3-benzoyl-2,7-dimethoxynaphthalene (1.0 mmol, 294 mg). After the reaction mixture was stirred at 273 K for 72 h, it was poured into ice-cold water (10 ml). The aqueous layer was extracted with CHCl_3_ (10 ml × 3). The combined extracts were washed with 2*M* aqueous NaOH followed by washing with brine. The organic layers thus obtained were dried over anhydrous MgSO_4_. The solvent was removed under reduced pressure to give cake (quant.). The crude product was purified by recrystallization from ethanol (34% yield). Colourless platelet single crystals suitable for X-ray diffraction analysis were obtained by repeated crystallization from a hexane/chloroform (1:1 *v*/*v*) solution. ^1^H NMR δ (400 MHz, CDCl_3_, p.p.m.); 3.60(3*H*, s), 3.82(3*H*, s), 6.88(1*H*, s), 7.23(1*H*, d, *J* = 8.4 Hz), 7.41–7.48(4*H*, m), 7.54–7.62(2*H*, m), 7.82–7.87(4*H*, m), 7.89(1*H*, d, *J* = 1.6 Hz), 7.92(1*H*, d, *J* = 9.2 Hz). ^13^C NMR δ (75 MHz, CDCl_3_, p.p.m.): 55.45, 56.31, 102.36, 110.99, 121.64, 123.35, 128.24, 128.63, 128.94, 129.51, 129.87, 130.26, 132.00, 133.04, 133.54, 134.06, 137.75, 137.94, 156.08, 156.39, 195.77, 197.72. IR (KBr); 1668(C═O), 1624, 1578, 1497(Ar, naphthalene) cm^-1^. HRMS (*m/z*); [*M* + H]^+^ Calcd for C_26_H_21_O_4_, 397.1440; found, 397.1444. M.p. = 429.4–431.8 K

### Refinement

All H atoms were found in a difference map and were subsequently refined as riding atoms, with C—H = 0.95 (aromatic) and 0.98 (methyl) Å, and with *U*_iso_(H) = 1.2*U*_eq_(C). Rigid bond restrains were applied to the *U^ij^* values of naphthalene ring (C31—C32) and benzene ring (C40—C41) [2 restrains with the DELU command in *SHELXL97*].

### Crystal data

**Table:**

C_26_H_20_O_4_	*Z* = 4
*M**_r_* = 396.42	*F*(000) = 832
Triclinic, *P*1	*D*_x_ = 1.301 Mg m^−^^3^
Hall symbol: -P 1	Melting point = 429.4–431.8 K
*a* = 8.42828 (15) Å	Cu *K*α radiation, λ = 1.54187 Å
*b* = 12.5953 (2) Å	Cell parameters from 27981 reflections
*c* = 20.0578 (4) Å	θ = 3.6–68.2°
α = 96.222 (1)°	µ = 0.71 mm^−^^1^
β = 99.688 (1)°	*T* = 193 K
γ = 102.727 (1)°	Platelet, colourless
*V* = 2023.76 (6) Å^3^	0.60 × 0.40 × 0.10 mm

### Data collection

**Table:**

Rigaku R-AXIS RAPID diffractometer	7263 independent reflections
Radiation source: rotating anode	5957 reflections with *I* > 2σ(*I*)
graphite	*R*_int_ = 0.031
Detector resolution: 10.000 pixels mm^-1^	θ_max_ = 68.2°, θ_min_ = 3.6°
ω scans	*h* = −10→10
Absorption correction: numerical (*NUMABS*; Higashi, 1999)	*k* = −15→15
*T*_min_ = 0.677, *T*_max_ = 0.933	*l* = −23→24
32431 measured reflections	

### Refinement

**Table:**

Refinement on *F*^2^	Secondary atom site location: difference Fourier map
Least-squares matrix: full	Hydrogen site location: inferred from neighbouring sites
*R*[*F*^2^ > 2σ(*F*^2^)] = 0.039	H-atom parameters constrained
*wR*(*F*^2^) = 0.115	*w* = 1/[σ^2^(*F*_o_^2^) + (0.0636*P*)^2^ + 0.2292*P*] where *P* = (*F*_o_^2^ + 2*F*_c_^2^)/3
*S* = 1.08	(Δ/σ)_max_ = 0.001
7263 reflections	Δρ_max_ = 0.27 e Å^−^^3^
546 parameters	Δρ_min_ = −0.21 e Å^−^^3^
2 restraints	Extinction correction: *SHELXL97* (Sheldrick, 2008), Fc^*^=kFc[1+0.001xFc^2^λ^3^/sin(2θ)]^-1/4^
Primary atom site location: structure-invariant direct methods	Extinction coefficient: 0.0067 (4)

### Special details

**Table:**

Geometry. All e.s.d.'s (except the e.s.d. in the dihedral angle between two l.s. planes) are estimated using the full covariance matrix. The cell e.s.d.'s are taken into account individually in the estimation of e.s.d.'s in distances, angles and torsion angles; correlations between e.s.d.'s in cell parameters are only used when they are defined by crystal symmetry. An approximate (isotropic) treatment of cell e.s.d.'s is used for estimating e.s.d.'s involving l.s. planes.
Refinement. Refinement of *F*^2^ against ALL reflections. The weighted *R*-factor *wR* and goodness of fit *S* are based on *F*^2^, conventional *R*-factors *R* are based on *F*, with *F* set to zero for negative *F*^2^. The threshold expression of *F*^2^ > σ(*F*^2^) is used only for calculating *R*-factors(gt) *etc*. and is not relevant to the choice of reflections for refinement. *R*-factors based on *F*^2^ are statistically about twice as large as those based on *F*, and *R*- factors based on ALL data will be even larger.

### Fractional atomic coordinates and isotropic or equivalent isotropic displacement parameters (Å^2^)

**Table:**

	*x*	*y*	*z*	*U*_iso_*/*U*_eq_	
O1	0.75620 (12)	1.12665 (8)	0.24000 (5)	0.0463 (3)	
O2	0.14926 (12)	0.65855 (8)	0.03150 (5)	0.0455 (3)	
O3	0.55140 (12)	0.85168 (9)	0.25446 (5)	0.0494 (3)	
O4	0.03981 (14)	0.81814 (11)	−0.11186 (6)	0.0629 (3)	
O5	0.39029 (14)	0.33247 (9)	0.27240 (6)	0.0541 (3)	
O6	0.39614 (13)	0.86845 (9)	0.44885 (5)	0.0511 (3)	
O7	0.38552 (13)	0.58829 (10)	0.22876 (6)	0.0564 (3)	
O8	0.36093 (17)	0.79742 (14)	0.60807 (6)	0.0856 (5)	
C1	0.63721 (16)	1.08595 (11)	0.18260 (7)	0.0382 (3)	
C2	0.58200 (17)	1.15268 (11)	0.13624 (8)	0.0423 (3)	
H2	0.6302	1.2297	0.1434	0.051*	
C3	0.45899 (17)	1.10584 (11)	0.08112 (7)	0.0410 (3)	
H3	0.4233	1.1509	0.0496	0.049*	
C4	0.38305 (16)	0.99224 (11)	0.06952 (7)	0.0364 (3)	
C5	0.25120 (16)	0.94529 (11)	0.01373 (7)	0.0383 (3)	
H5	0.2160	0.9910	−0.0175	0.046*	
C6	0.17237 (16)	0.83613 (11)	0.00312 (7)	0.0377 (3)	
C7	0.23026 (16)	0.76713 (11)	0.04875 (7)	0.0367 (3)	
C8	0.35891 (15)	0.80916 (11)	0.10324 (7)	0.0357 (3)	
H8	0.3957	0.7617	0.1328	0.043*	
C9	0.43812 (15)	0.92381 (11)	0.11589 (7)	0.0340 (3)	
C10	0.57067 (15)	0.97328 (11)	0.17229 (7)	0.0350 (3)	
C11	0.63953 (16)	0.90486 (11)	0.22145 (7)	0.0354 (3)	
C12	0.81623 (15)	0.89971 (10)	0.22638 (7)	0.0342 (3)	
C13	0.91210 (17)	0.94624 (12)	0.18213 (7)	0.0419 (3)	
H13	0.8666	0.9849	0.1486	0.050*	
C14	1.07400 (18)	0.93639 (14)	0.18677 (8)	0.0505 (4)	
H14	1.1390	0.9682	0.1563	0.061*	
C15	1.14103 (18)	0.88071 (14)	0.23533 (8)	0.0519 (4)	
H15	1.2518	0.8736	0.2382	0.062*	
C16	1.04694 (18)	0.83527 (14)	0.27983 (9)	0.0526 (4)	
H16	1.0936	0.7976	0.3137	0.063*	
C17	0.88488 (17)	0.84427 (12)	0.27543 (8)	0.0437 (3)	
H17	0.8205	0.8124	0.3061	0.052*	
C18	0.02760 (17)	0.79442 (12)	−0.05542 (7)	0.0414 (3)	
C19	−0.13454 (16)	0.73366 (10)	−0.04292 (7)	0.0376 (3)	
C20	−0.17214 (18)	0.73856 (12)	0.02197 (8)	0.0448 (3)	
H20	−0.0896	0.7760	0.0606	0.054*	
C21	−0.3297 (2)	0.68907 (14)	0.03039 (10)	0.0581 (4)	
H21	−0.3558	0.6935	0.0747	0.070*	
C22	−0.4493 (2)	0.63315 (14)	−0.02576 (11)	0.0633 (5)	
H22	−0.5573	0.5988	−0.0199	0.076*	
C23	−0.4121 (2)	0.62724 (12)	−0.08983 (11)	0.0590 (5)	
H23	−0.4945	0.5884	−0.1282	0.071*	
C24	−0.25615 (18)	0.67716 (11)	−0.09900 (8)	0.0464 (4)	
H24	−0.2316	0.6730	−0.1436	0.056*	
C25	0.8244 (2)	1.24308 (12)	0.25537 (9)	0.0547 (4)	
H25A	0.9051	1.2604	0.2987	0.066*	
H25B	0.7351	1.2804	0.2592	0.066*	
H25C	0.8798	1.2682	0.2187	0.066*	
C26	0.1872 (2)	0.58516 (12)	0.07768 (9)	0.0529 (4)	
H26A	0.3027	0.5808	0.0801	0.064*	
H26B	0.1132	0.5118	0.0614	0.064*	
H26C	0.1718	0.6124	0.1232	0.064*	
C27	0.36484 (18)	0.39304 (13)	0.32852 (8)	0.0460 (3)	
C28	0.3038 (2)	0.34626 (14)	0.38244 (9)	0.0543 (4)	
H28	0.2800	0.2688	0.3816	0.065*	
C29	0.2792 (2)	0.41289 (14)	0.43577 (9)	0.0550 (4)	
H29	0.2397	0.3808	0.4724	0.066*	
C30	0.31049 (17)	0.52816 (13)	0.43836 (8)	0.0456 (3)	
C31	0.28001 (18)	0.59751 (14)	0.49256 (8)	0.0497 (4)	
H31	0.2393	0.5657	0.5291	0.060*	
C32	0.30714 (17)	0.70823 (13)	0.49412 (7)	0.0456 (3)	
C33	0.37117 (17)	0.75666 (13)	0.44010 (7)	0.0434 (3)	
C34	0.40361 (16)	0.69303 (12)	0.38659 (7)	0.0411 (3)	
H34	0.4465	0.7267	0.3511	0.049*	
C35	0.37334 (16)	0.57641 (12)	0.38400 (7)	0.0401 (3)	
C36	0.40236 (16)	0.50614 (12)	0.32941 (7)	0.0412 (3)	
C37	0.47086 (17)	0.55206 (11)	0.27136 (7)	0.0400 (3)	
C38	0.64577 (17)	0.55189 (11)	0.26701 (7)	0.0406 (3)	
C39	0.75209 (19)	0.52482 (14)	0.31930 (9)	0.0535 (4)	
H39	0.7149	0.5068	0.3598	0.064*	
C40	0.9143 (2)	0.52407 (15)	0.31253 (11)	0.0673 (5)	
H40	0.9881	0.5068	0.3487	0.081*	
C41	0.9670 (2)	0.54832 (16)	0.25342 (12)	0.0731 (6)	
H41	1.0764	0.5462	0.2484	0.088*	
C42	0.8617 (2)	0.57558 (17)	0.20157 (11)	0.0698 (5)	
H42	0.8984	0.5921	0.1608	0.084*	
C43	0.70313 (19)	0.57898 (13)	0.20861 (8)	0.0517 (4)	
H43	0.6322	0.6001	0.1731	0.062*	
C44	0.26992 (19)	0.77897 (14)	0.55201 (8)	0.0515 (4)	
C45	0.11741 (17)	0.82003 (12)	0.54022 (7)	0.0426 (3)	
C46	0.00223 (18)	0.78735 (12)	0.47869 (8)	0.0456 (3)	
H46	0.0234	0.7406	0.4425	0.055*	
C47	−0.1424 (2)	0.82298 (14)	0.47039 (9)	0.0545 (4)	
H47	−0.2213	0.8000	0.4286	0.065*	
C48	−0.1730 (2)	0.89198 (14)	0.52258 (9)	0.0570 (4)	
H48	−0.2727	0.9164	0.5166	0.068*	
C49	−0.0592 (2)	0.92543 (13)	0.58321 (9)	0.0542 (4)	
H49	−0.0804	0.9732	0.6189	0.065*	
C50	0.08573 (19)	0.88984 (12)	0.59252 (8)	0.0479 (4)	
H50	0.1637	0.9129	0.6345	0.057*	
C51	0.3316 (2)	0.21502 (13)	0.26385 (9)	0.0578 (4)	
H51A	0.3462	0.1827	0.2193	0.069*	
H51B	0.2136	0.1959	0.2661	0.069*	
H51C	0.3947	0.1862	0.3003	0.069*	
C52	0.46383 (19)	0.92637 (13)	0.39871 (8)	0.0499 (4)	
H52A	0.4778	1.0056	0.4119	0.060*	
H52B	0.3883	0.9019	0.3542	0.060*	
H52C	0.5718	0.9114	0.3956	0.060*	

### Atomic displacement parameters (Å^2^)

**Table:**

	*U*^11^	*U*^22^	*U*^33^	*U*^12^	*U*^13^	*U*^23^
O1	0.0494 (6)	0.0427 (5)	0.0421 (6)	0.0103 (4)	0.0006 (5)	0.0016 (4)
O2	0.0443 (5)	0.0388 (5)	0.0503 (6)	0.0099 (4)	−0.0024 (5)	0.0124 (4)
O3	0.0365 (5)	0.0686 (7)	0.0494 (6)	0.0138 (5)	0.0130 (5)	0.0271 (5)
O4	0.0529 (7)	0.0952 (9)	0.0393 (6)	0.0091 (6)	0.0066 (5)	0.0267 (6)
O5	0.0627 (7)	0.0495 (6)	0.0495 (7)	0.0096 (5)	0.0141 (5)	0.0088 (5)
O6	0.0549 (6)	0.0536 (6)	0.0465 (6)	0.0110 (5)	0.0171 (5)	0.0081 (5)
O7	0.0478 (6)	0.0831 (8)	0.0468 (6)	0.0245 (6)	0.0108 (5)	0.0267 (6)
O8	0.0697 (8)	0.1516 (14)	0.0404 (7)	0.0559 (9)	−0.0025 (6)	−0.0036 (8)
C1	0.0345 (7)	0.0443 (7)	0.0382 (8)	0.0135 (6)	0.0092 (6)	0.0056 (6)
C2	0.0421 (8)	0.0378 (7)	0.0488 (9)	0.0120 (6)	0.0101 (6)	0.0082 (6)
C3	0.0403 (7)	0.0419 (7)	0.0466 (8)	0.0165 (6)	0.0105 (6)	0.0159 (6)
C4	0.0330 (7)	0.0430 (7)	0.0385 (7)	0.0145 (5)	0.0116 (6)	0.0122 (6)
C5	0.0360 (7)	0.0466 (7)	0.0379 (8)	0.0156 (6)	0.0092 (6)	0.0167 (6)
C6	0.0325 (7)	0.0474 (7)	0.0360 (7)	0.0126 (6)	0.0074 (6)	0.0120 (6)
C7	0.0324 (7)	0.0404 (7)	0.0398 (8)	0.0112 (5)	0.0082 (6)	0.0105 (6)
C8	0.0326 (7)	0.0414 (7)	0.0386 (7)	0.0153 (5)	0.0086 (6)	0.0136 (6)
C9	0.0293 (6)	0.0419 (7)	0.0362 (7)	0.0143 (5)	0.0114 (5)	0.0095 (6)
C10	0.0309 (6)	0.0425 (7)	0.0358 (7)	0.0143 (5)	0.0096 (5)	0.0086 (6)
C11	0.0329 (7)	0.0412 (7)	0.0324 (7)	0.0090 (5)	0.0071 (5)	0.0062 (6)
C12	0.0312 (7)	0.0370 (6)	0.0328 (7)	0.0086 (5)	0.0028 (5)	0.0029 (5)
C13	0.0374 (7)	0.0555 (8)	0.0363 (8)	0.0154 (6)	0.0089 (6)	0.0105 (6)
C14	0.0375 (8)	0.0733 (10)	0.0430 (9)	0.0149 (7)	0.0130 (6)	0.0079 (7)
C15	0.0335 (7)	0.0730 (11)	0.0485 (9)	0.0193 (7)	0.0032 (7)	0.0011 (8)
C16	0.0420 (8)	0.0635 (10)	0.0531 (10)	0.0208 (7)	−0.0020 (7)	0.0143 (8)
C17	0.0378 (7)	0.0485 (8)	0.0452 (8)	0.0103 (6)	0.0051 (6)	0.0138 (6)
C18	0.0410 (8)	0.0491 (8)	0.0367 (8)	0.0145 (6)	0.0062 (6)	0.0122 (6)
C19	0.0382 (7)	0.0366 (7)	0.0393 (8)	0.0131 (5)	0.0028 (6)	0.0108 (6)
C20	0.0411 (8)	0.0520 (8)	0.0445 (8)	0.0149 (6)	0.0061 (6)	0.0169 (7)
C21	0.0502 (9)	0.0697 (11)	0.0676 (11)	0.0217 (8)	0.0212 (8)	0.0374 (9)
C22	0.0401 (9)	0.0490 (9)	0.1035 (16)	0.0077 (7)	0.0110 (9)	0.0343 (10)
C23	0.0454 (9)	0.0394 (8)	0.0824 (13)	0.0080 (7)	−0.0092 (9)	0.0054 (8)
C24	0.0477 (8)	0.0403 (7)	0.0487 (9)	0.0159 (6)	−0.0034 (7)	0.0046 (6)
C25	0.0589 (10)	0.0460 (8)	0.0514 (10)	0.0046 (7)	0.0043 (8)	0.0009 (7)
C26	0.0506 (9)	0.0443 (8)	0.0618 (10)	0.0093 (7)	0.0004 (8)	0.0204 (7)
C27	0.0418 (8)	0.0532 (8)	0.0427 (8)	0.0097 (6)	0.0074 (6)	0.0120 (7)
C28	0.0579 (10)	0.0518 (9)	0.0534 (10)	0.0073 (7)	0.0128 (8)	0.0182 (8)
C29	0.0557 (9)	0.0646 (10)	0.0486 (10)	0.0096 (8)	0.0173 (8)	0.0255 (8)
C30	0.0396 (8)	0.0593 (9)	0.0396 (8)	0.0099 (6)	0.0090 (6)	0.0168 (7)
C31	0.0450 (8)	0.0716 (10)	0.0359 (8)	0.0125 (7)	0.0127 (6)	0.0192 (7)
C32	0.0381 (7)	0.0643 (9)	0.0355 (8)	0.0134 (7)	0.0068 (6)	0.0112 (7)
C33	0.0337 (7)	0.0566 (9)	0.0385 (8)	0.0082 (6)	0.0051 (6)	0.0106 (7)
C34	0.0327 (7)	0.0549 (8)	0.0359 (8)	0.0073 (6)	0.0082 (6)	0.0124 (6)
C35	0.0298 (7)	0.0551 (8)	0.0350 (7)	0.0077 (6)	0.0048 (5)	0.0128 (6)
C36	0.0337 (7)	0.0517 (8)	0.0377 (8)	0.0085 (6)	0.0053 (6)	0.0115 (6)
C37	0.0364 (7)	0.0461 (7)	0.0357 (8)	0.0082 (6)	0.0043 (6)	0.0066 (6)
C38	0.0364 (7)	0.0409 (7)	0.0412 (8)	0.0047 (6)	0.0066 (6)	0.0036 (6)
C39	0.0413 (8)	0.0607 (9)	0.0565 (10)	0.0108 (7)	0.0040 (7)	0.0125 (8)
C40	0.0405 (9)	0.0676 (11)	0.0880 (14)	0.0139 (8)	−0.0037 (9)	0.0113 (10)
C41	0.0414 (9)	0.0769 (12)	0.1002 (16)	0.0101 (8)	0.0242 (10)	0.0030 (11)
C42	0.0481 (10)	0.0860 (13)	0.0727 (13)	0.0028 (9)	0.0272 (9)	0.0055 (10)
C43	0.0436 (8)	0.0582 (9)	0.0490 (9)	0.0013 (7)	0.0127 (7)	0.0065 (7)
C44	0.0449 (8)	0.0732 (11)	0.0368 (8)	0.0136 (7)	0.0083 (7)	0.0110 (7)
C45	0.0408 (7)	0.0479 (8)	0.0387 (8)	0.0051 (6)	0.0105 (6)	0.0134 (6)
C46	0.0453 (8)	0.0500 (8)	0.0409 (8)	0.0084 (6)	0.0085 (6)	0.0110 (6)
C47	0.0481 (9)	0.0646 (10)	0.0484 (9)	0.0128 (7)	0.0010 (7)	0.0140 (8)
C48	0.0516 (9)	0.0609 (10)	0.0642 (11)	0.0210 (8)	0.0129 (8)	0.0171 (8)
C49	0.0586 (10)	0.0486 (9)	0.0582 (10)	0.0137 (7)	0.0181 (8)	0.0089 (7)
C50	0.0474 (8)	0.0506 (8)	0.0424 (8)	0.0039 (7)	0.0095 (7)	0.0085 (7)
C51	0.0624 (10)	0.0501 (9)	0.0607 (11)	0.0146 (8)	0.0095 (8)	0.0107 (8)
C52	0.0500 (9)	0.0558 (9)	0.0451 (9)	0.0117 (7)	0.0106 (7)	0.0135 (7)

### Geometric parameters (Å, °)

**Table:**

O1—C1	1.3606 (16)	C25—H25A	0.9800
O1—C25	1.4306 (17)	C25—H25B	0.9800
O2—C7	1.3647 (16)	C25—H25C	0.9800
O2—C26	1.4251 (17)	C26—H26A	0.9800
O3—C11	1.2172 (16)	C26—H26B	0.9800
O4—C18	1.2168 (17)	C26—H26C	0.9800
O5—C27	1.3646 (18)	C27—C36	1.387 (2)
O5—C51	1.4340 (18)	C27—C28	1.405 (2)
O6—C33	1.3641 (18)	C28—C29	1.360 (2)
O6—C52	1.4276 (18)	C28—H28	0.9500
O7—C37	1.2157 (17)	C29—C30	1.411 (2)
O8—C44	1.2170 (18)	C29—H29	0.9500
C1—C10	1.3848 (19)	C30—C31	1.414 (2)
C1—C2	1.408 (2)	C30—C35	1.426 (2)
C2—C3	1.360 (2)	C31—C32	1.359 (2)
C2—H2	0.9500	C31—H31	0.9500
C3—C4	1.4090 (19)	C32—C33	1.426 (2)
C3—H3	0.9500	C32—C44	1.504 (2)
C4—C5	1.4090 (19)	C33—C34	1.370 (2)
C4—C9	1.4242 (18)	C34—C35	1.428 (2)
C5—C6	1.3652 (19)	C34—H34	0.9500
C5—H5	0.9500	C35—C36	1.420 (2)
C6—C7	1.4282 (19)	C36—C37	1.500 (2)
C6—C18	1.4984 (18)	C37—C38	1.4918 (19)
C7—C8	1.3687 (18)	C38—C39	1.384 (2)
C8—C9	1.4261 (18)	C38—C43	1.388 (2)
C8—H8	0.9500	C39—C40	1.399 (2)
C9—C10	1.4252 (18)	C39—H39	0.9500
C10—C11	1.5045 (19)	C40—C41	1.376 (3)
C11—C12	1.4921 (18)	C40—H40	0.9500
C12—C17	1.3875 (19)	C41—C42	1.374 (3)
C12—C13	1.3892 (19)	C41—H41	0.9500
C13—C14	1.386 (2)	C42—C43	1.377 (2)
C13—H13	0.9500	C42—H42	0.9500
C14—C15	1.377 (2)	C43—H43	0.9500
C14—H14	0.9500	C44—C45	1.481 (2)
C15—C16	1.379 (2)	C45—C50	1.394 (2)
C15—H15	0.9500	C45—C46	1.395 (2)
C16—C17	1.384 (2)	C46—C47	1.380 (2)
C16—H16	0.9500	C46—H46	0.9500
C17—H17	0.9500	C47—C48	1.382 (2)
C18—C19	1.4872 (19)	C47—H47	0.9500
C19—C20	1.389 (2)	C48—C49	1.377 (2)
C19—C24	1.3927 (19)	C48—H48	0.9500
C20—C21	1.384 (2)	C49—C50	1.382 (2)
C20—H20	0.9500	C49—H49	0.9500
C21—C22	1.384 (3)	C50—H50	0.9500
C21—H21	0.9500	C51—H51A	0.9800
C22—C23	1.371 (3)	C51—H51B	0.9800
C22—H22	0.9500	C51—H51C	0.9800
C23—C24	1.378 (2)	C52—H52A	0.9800
C23—H23	0.9500	C52—H52B	0.9800
C24—H24	0.9500	C52—H52C	0.9800
			
C1—O1—C25	118.57 (11)	H26A—C26—H26C	109.5
C7—O2—C26	117.91 (11)	H26B—C26—H26C	109.5
C27—O5—C51	118.04 (12)	O5—C27—C36	115.63 (13)
C33—O6—C52	118.14 (11)	O5—C27—C28	123.42 (14)
O1—C1—C10	115.76 (12)	C36—C27—C28	120.96 (14)
O1—C1—C2	123.07 (12)	C29—C28—C27	119.40 (15)
C10—C1—C2	121.16 (13)	C29—C28—H28	120.3
C3—C2—C1	119.35 (13)	C27—C28—H28	120.3
C3—C2—H2	120.3	C28—C29—C30	122.05 (15)
C1—C2—H2	120.3	C28—C29—H29	119.0
C2—C3—C4	121.78 (13)	C30—C29—H29	119.0
C2—C3—H3	119.1	C29—C30—C31	122.31 (14)
C4—C3—H3	119.1	C29—C30—C35	118.90 (14)
C5—C4—C3	121.30 (12)	C31—C30—C35	118.78 (14)
C5—C4—C9	119.27 (12)	C32—C31—C30	122.09 (14)
C3—C4—C9	119.41 (12)	C32—C31—H31	119.0
C6—C5—C4	122.06 (12)	C30—C31—H31	119.0
C6—C5—H5	119.0	C31—C32—C33	119.19 (14)
C4—C5—H5	119.0	C31—C32—C44	120.49 (14)
C5—C6—C7	118.58 (12)	C33—C32—C44	120.32 (14)
C5—C6—C18	118.45 (12)	O6—C33—C34	125.97 (13)
C7—C6—C18	122.97 (12)	O6—C33—C32	113.10 (13)
O2—C7—C8	124.78 (12)	C34—C33—C32	120.93 (14)
O2—C7—C6	113.96 (11)	C33—C34—C35	120.31 (13)
C8—C7—C6	121.23 (12)	C33—C34—H34	119.8
C7—C8—C9	120.42 (12)	C35—C34—H34	119.8
C7—C8—H8	119.8	C36—C35—C30	118.47 (13)
C9—C8—H8	119.8	C36—C35—C34	122.84 (13)
C4—C9—C10	118.28 (12)	C30—C35—C34	118.69 (13)
C4—C9—C8	118.39 (12)	C27—C36—C35	120.16 (13)
C10—C9—C8	123.33 (12)	C27—C36—C37	118.84 (13)
C1—C10—C9	119.93 (12)	C35—C36—C37	120.99 (13)
C1—C10—C11	119.13 (12)	O7—C37—C38	120.84 (13)
C9—C10—C11	120.94 (12)	O7—C37—C36	120.69 (13)
O3—C11—C12	120.80 (12)	C38—C37—C36	118.47 (12)
O3—C11—C10	120.73 (12)	C39—C38—C43	119.07 (14)
C12—C11—C10	118.42 (11)	C39—C38—C37	121.92 (14)
C17—C12—C13	119.27 (12)	C43—C38—C37	119.01 (13)
C17—C12—C11	118.88 (12)	C38—C39—C40	119.94 (17)
C13—C12—C11	121.83 (12)	C38—C39—H39	120.0
C14—C13—C12	120.13 (14)	C40—C39—H39	120.0
C14—C13—H13	119.9	C41—C40—C39	119.94 (18)
C12—C13—H13	119.9	C41—C40—H40	120.0
C15—C14—C13	120.28 (15)	C39—C40—H40	120.0
C15—C14—H14	119.9	C42—C41—C40	120.14 (16)
C13—C14—H14	119.9	C42—C41—H41	119.9
C14—C15—C16	119.83 (14)	C40—C41—H41	119.9
C14—C15—H15	120.1	C41—C42—C43	120.15 (18)
C16—C15—H15	120.1	C41—C42—H42	119.9
C15—C16—C17	120.33 (14)	C43—C42—H42	119.9
C15—C16—H16	119.8	C42—C43—C38	120.70 (17)
C17—C16—H16	119.8	C42—C43—H43	119.6
C16—C17—C12	120.16 (14)	C38—C43—H43	119.6
C16—C17—H17	119.9	O8—C44—C45	121.31 (15)
C12—C17—H17	119.9	O8—C44—C32	119.79 (15)
O4—C18—C19	120.28 (13)	C45—C44—C32	118.83 (13)
O4—C18—C6	119.60 (13)	C50—C45—C46	119.36 (14)
C19—C18—C6	119.86 (12)	C50—C45—C44	119.28 (14)
C20—C19—C24	119.30 (14)	C46—C45—C44	121.32 (14)
C20—C19—C18	122.06 (12)	C47—C46—C45	119.98 (15)
C24—C19—C18	118.46 (13)	C47—C46—H46	120.0
C21—C20—C19	120.10 (15)	C45—C46—H46	120.0
C21—C20—H20	120.0	C46—C47—C48	120.27 (15)
C19—C20—H20	120.0	C46—C47—H47	119.9
C22—C21—C20	119.99 (17)	C48—C47—H47	119.9
C22—C21—H21	120.0	C49—C48—C47	120.08 (15)
C20—C21—H21	120.0	C49—C48—H48	120.0
C23—C22—C21	120.04 (16)	C47—C48—H48	120.0
C23—C22—H22	120.0	C48—C49—C50	120.36 (15)
C21—C22—H22	120.0	C48—C49—H49	119.8
C22—C23—C24	120.54 (16)	C50—C49—H49	119.8
C22—C23—H23	119.7	C49—C50—C45	119.94 (15)
C24—C23—H23	119.7	C49—C50—H50	120.0
C23—C24—C19	120.02 (16)	C45—C50—H50	120.0
C23—C24—H24	120.0	O5—C51—H51A	109.5
C19—C24—H24	120.0	O5—C51—H51B	109.5
O1—C25—H25A	109.5	H51A—C51—H51B	109.5
O1—C25—H25B	109.5	O5—C51—H51C	109.5
H25A—C25—H25B	109.5	H51A—C51—H51C	109.5
O1—C25—H25C	109.5	H51B—C51—H51C	109.5
H25A—C25—H25C	109.5	O6—C52—H52A	109.5
H25B—C25—H25C	109.5	O6—C52—H52B	109.5
O2—C26—H26A	109.5	H52A—C52—H52B	109.5
O2—C26—H26B	109.5	O6—C52—H52C	109.5
H26A—C26—H26B	109.5	H52A—C52—H52C	109.5
O2—C26—H26C	109.5	H52B—C52—H52C	109.5
			
C25—O1—C1—C10	−177.07 (12)	C51—O5—C27—C36	−171.18 (13)
C25—O1—C1—C2	2.29 (19)	C51—O5—C27—C28	8.4 (2)
O1—C1—C2—C3	−178.06 (12)	O5—C27—C28—C29	−178.70 (14)
C10—C1—C2—C3	1.3 (2)	C36—C27—C28—C29	0.9 (2)
C1—C2—C3—C4	1.0 (2)	C27—C28—C29—C30	1.1 (2)
C2—C3—C4—C5	177.43 (13)	C28—C29—C30—C31	177.89 (16)
C2—C3—C4—C9	−1.2 (2)	C28—C29—C30—C35	−1.4 (2)
C3—C4—C5—C6	−177.64 (13)	C29—C30—C31—C32	−178.62 (14)
C9—C4—C5—C6	0.99 (19)	C35—C30—C31—C32	0.7 (2)
C4—C5—C6—C7	−2.4 (2)	C30—C31—C32—C33	−1.1 (2)
C4—C5—C6—C18	176.84 (12)	C30—C31—C32—C44	178.77 (14)
C26—O2—C7—C8	7.8 (2)	C52—O6—C33—C34	−0.7 (2)
C26—O2—C7—C6	−173.92 (13)	C52—O6—C33—C32	178.53 (12)
C5—C6—C7—O2	−176.53 (12)	C31—C32—C33—O6	−178.54 (12)
C18—C6—C7—O2	4.24 (18)	C44—C32—C33—O6	1.55 (19)
C5—C6—C7—C8	1.8 (2)	C31—C32—C33—C34	0.8 (2)
C18—C6—C7—C8	−177.42 (12)	C44—C32—C33—C34	−179.15 (13)
O2—C7—C8—C9	178.41 (12)	O6—C33—C34—C35	179.31 (12)
C6—C7—C8—C9	0.26 (19)	C32—C33—C34—C35	0.1 (2)
C5—C4—C9—C10	−179.44 (11)	C29—C30—C35—C36	−0.2 (2)
C3—C4—C9—C10	−0.78 (18)	C31—C30—C35—C36	−179.48 (13)
C5—C4—C9—C8	1.10 (18)	C29—C30—C35—C34	179.53 (13)
C3—C4—C9—C8	179.75 (12)	C31—C30—C35—C34	0.2 (2)
C7—C8—C9—C4	−1.69 (18)	C33—C34—C35—C36	179.10 (13)
C7—C8—C9—C10	178.87 (12)	C33—C34—C35—C30	−0.6 (2)
O1—C1—C10—C9	176.13 (11)	O5—C27—C36—C35	177.17 (12)
C2—C1—C10—C9	−3.24 (19)	C28—C27—C36—C35	−2.4 (2)
O1—C1—C10—C11	−3.54 (17)	O5—C27—C36—C37	−1.77 (19)
C2—C1—C10—C11	177.08 (12)	C28—C27—C36—C37	178.60 (14)
C4—C9—C10—C1	2.95 (18)	C30—C35—C36—C27	2.1 (2)
C8—C9—C10—C1	−177.61 (12)	C34—C35—C36—C27	−177.65 (13)
C4—C9—C10—C11	−177.38 (11)	C30—C35—C36—C37	−179.02 (12)
C8—C9—C10—C11	2.06 (19)	C34—C35—C36—C37	1.3 (2)
C1—C10—C11—O3	119.31 (15)	C27—C36—C37—O7	106.69 (17)
C9—C10—C11—O3	−60.36 (18)	C35—C36—C37—O7	−72.25 (19)
C1—C10—C11—C12	−63.44 (16)	C27—C36—C37—C38	−73.11 (17)
C9—C10—C11—C12	116.89 (13)	C35—C36—C37—C38	107.95 (15)
O3—C11—C12—C17	−7.9 (2)	O7—C37—C38—C39	171.32 (14)
C10—C11—C12—C17	174.81 (12)	C36—C37—C38—C39	−8.9 (2)
O3—C11—C12—C13	170.31 (13)	O7—C37—C38—C43	−9.1 (2)
C10—C11—C12—C13	−6.93 (19)	C36—C37—C38—C43	170.69 (13)
C17—C12—C13—C14	0.5 (2)	C43—C38—C39—C40	−0.6 (2)
C11—C12—C13—C14	−177.72 (13)	C37—C38—C39—C40	178.99 (14)
C12—C13—C14—C15	−0.2 (2)	C38—C39—C40—C41	−1.2 (3)
C13—C14—C15—C16	−0.5 (2)	C39—C40—C41—C42	1.4 (3)
C14—C15—C16—C17	0.7 (2)	C40—C41—C42—C43	0.1 (3)
C15—C16—C17—C12	−0.4 (2)	C41—C42—C43—C38	−1.9 (3)
C13—C12—C17—C16	−0.3 (2)	C39—C38—C43—C42	2.1 (2)
C11—C12—C17—C16	178.04 (13)	C37—C38—C43—C42	−177.46 (15)
C5—C6—C18—O4	48.67 (19)	C31—C32—C44—O8	73.7 (2)
C7—C6—C18—O4	−132.11 (15)	C33—C32—C44—O8	−106.41 (19)
C5—C6—C18—C19	−125.45 (14)	C31—C32—C44—C45	−103.19 (17)
C7—C6—C18—C19	53.78 (18)	C33—C32—C44—C45	76.72 (19)
O4—C18—C19—C20	−156.43 (15)	O8—C44—C45—C50	6.2 (2)
C6—C18—C19—C20	17.6 (2)	C32—C44—C45—C50	−177.02 (14)
O4—C18—C19—C24	18.6 (2)	O8—C44—C45—C46	−171.66 (17)
C6—C18—C19—C24	−167.32 (12)	C32—C44—C45—C46	5.2 (2)
C24—C19—C20—C21	−1.0 (2)	C50—C45—C46—C47	−0.8 (2)
C18—C19—C20—C21	174.04 (13)	C44—C45—C46—C47	177.00 (14)
C19—C20—C21—C22	1.0 (2)	C45—C46—C47—C48	0.7 (2)
C20—C21—C22—C23	−0.3 (2)	C46—C47—C48—C49	−0.1 (3)
C21—C22—C23—C24	−0.3 (2)	C47—C48—C49—C50	−0.4 (2)
C22—C23—C24—C19	0.4 (2)	C48—C49—C50—C45	0.2 (2)
C20—C19—C24—C23	0.3 (2)	C46—C45—C50—C49	0.4 (2)
C18—C19—C24—C23	−174.90 (13)	C44—C45—C50—C49	−177.50 (14)

### Hydrogen-bond geometry (Å, °)

**Table:**

Cg1 and Cg2 are the centroids of the C19–C24 and C4–C9 rings, respectively.

**Table:**

*D*—H···*A*	*D*—H	H···*A*	*D*···*A*	*D*—H···*A*
C15—H15···O3^i^	0.95	2.57	3.5191 (19)	176
C25—H25C···O4^ii^	0.98	2.56	3.348 (2)	138
C51—H51C···O8^iii^	0.98	2.47	3.371 (2)	152
C3—H3···Cg1^iv^	0.95	2.59	3.416 (11)	145
C14—H14···Cg2^v^	0.95	2.86	3.578 (9)	133

Symmetry codes: (i) *x*+1, *y*, *z*; (ii) −*x*+1, −*y*+2, −*z*; (iii) −*x*+1, −*y*+1, −*z*+1; (iv) −*x*, −*y*+2, −*z*; (v) *x*−1, *y*, *z*.
